# BAG3 protects Bovine Papillomavirus type 1-transformed equine fibroblasts against pro-death signals

**DOI:** 10.1186/1297-9716-44-61

**Published:** 2013-07-22

**Authors:** Roberta Cotugno, Dario Gallotta, Morena d’Avenia, Annunziata Corteggio, Gennaro Altamura, Franco Roperto, Maria Antonietta Belisario, Giuseppe Borzacchiello

**Affiliations:** 1 Department of Pharmacy, University of Salerno, Via Giovanni Paolo II n.132, 84084, Fisciano, Salerno, Italy; 2 Department of Veterinary Medicine and Animal Production, University of Naples, Federico II- Via F. Delpino, 80137, Napoli, Italy

## Abstract

In human cancer cells, BAG3 protein is known to sustain cell survival. Here, for the first time, we demonstrate the expression of BAG3 protein both in equine sarcoids in vivo and in EqS04b cells, a sarcoid-derived fully transformed cell line harbouring bovine papilloma virus (BPV)-1 genome. Evidence of a possible involvement of BAG3 in equine sarcoid carcinogenesis was obtained by immunohistochemistry analysis of tumour samples. We found that most tumour samples stained positive for BAG3, even though to a different grade, while normal dermal fibroblasts from healthy horses displayed very weak staining pattern for BAG3 expression. By siRNA technology, we demonstrate in EqS04b the role of BAG3 in counteracting basal as well as chemical-triggered pro-death signals. BAG3 down-modulation was indeed shown to promote cell death and cell cycle arrest in G_0_/G_1_. In addition, we found that BAG3 silencing sensitized EqS04b cells to phenethylisothiocyanate (PEITC), a promising cancer chemopreventive/chemotherapeutic agent present in edible cruciferous vegetables. Notably, such a pro-survival role of BAG3 was less marked in E. Derm cells, an equine BPV-negative fibroblast cell line taken as a normal counterpart. Altogether our findings might suggest a mutual cooperation between BAG3 and viral oncoproteins to sustain cell survival.

## Introduction

Sarcoids are the most common dermatological neoplasms affecting equids [1]. These neoplasms are benign lesions of fibroblastic origin, that often occur at sites of previous injury or scarring; they are locally aggressive and invasive, but rarely metastasize [2]. Histologically, the sarcoids are characterized by dermal proliferation of fibroblasts, forming whorls and epidermal hyperplasia. Although the pathology of this equine neoplasm is not completely understood, bovine papillomavirus (BPV) is considered to be the etiological agent. BPV type 1 and type 2 (BPV-1/-2) are non-enveloped, double stranded, DNA viruses, which commonly infect their natural host. However, BPV-1, and less commonly BPV-2, have been detected in sarcoids in different geographic areas of the world [3]. The major transforming product of BPV is E5, a very small membrane-associated protein with potent biological activities. It has been well recognized that E5 oncoprotein plays a key role during the development of BPV-induced tumours [4]. E5 oncogene is transcriptionally active and the protein is expressed in the neoplastic fibroblasts and overlying hyperplastic epidermis of sarcoids, where the BPV completes its life cycle producing virion particles [5,6].

Apoptosis is a non-inflammatory death process activated by cells to escape from viral infections, since cell death does not allow a complete viral replication cycle. Therefore, virus, in turn, can activate signalling pathways to prevent host cellular death [7]. The anti-apoptotic cellular machinery includes several proteins, among which the BAG family molecular chaperone regulator 3 (BAG3). BAG3, a member of a family of co-chaperones, shares the conserved BAG domain by which it interacts with heat shock proteins and other partners [8]. BAG3 is overexpressed in several human tumours, where it sustains cell survival through down-modulation of apoptosis [8,9]. *bag3* gene expression may also be induced in normal cells by several stressful agents [8,10,11] and virus. Recent studies have demonstrated that BAG3 plays an important role in the interaction of HIV-1 with host cells, thus controlling virus infection [12,13]. Indeed, in HIV-1 infected human microglia cells, BAG3 overexpression sustains cell survival by blocking caspase-3 activation and interfering with Akt proteasome translocation. Moreover, it was shown that BAG3 silencing inhibits Varicella Zoster Virus replication [14]. In Epstein Barr virus (EBV)-infected fibroblasts, apoptosis inhibition and a higher resistance to cytotoxic drugs has been associated to positive modulation of BAG3 and HSP70 expression by EBNA3A oncoprotein, a member of EBV nuclear antigens [15].

In the present study we focused on a possible involvement of BAG3 in equine sarcoid carcinogenesis. We demonstrate that BAG3 is selectively expressed in sarcoid tumour samples and highlight its pro-survival role in EqS04b, a sarcoid-derived cell line harbouring BPV-1 genome.

## Material and methods

### Reagents and antibodies

Fetal Bovine Serum (FBS) was from GIBCO (Life Technologies, Grand Island, NY, USA). All the other reagents were from Sigma-Aldrich (St. Louis, MO, USA). Anti-BAG3 (TOS-2) and anti-BAG3 (AC-1 clone) (BIOUNIVERSA, Fisciano, SA, Italy), anti-GAPDH (mouse monoclonal, sc-32233), anti-α tubulin (mouse monoclonal, sc-32293), anti-β actin (mouse monoclonal, sc-47778) from Santa Cruz Biotechnology (Santa Cruz, CA, USA); appropriate peroxidase-conjugated secondary antibodies were from Jackson ImmunoResearch (Baltimore, PA, USA).

### Tumour samples

A total of 15 equine sarcoids of different clinical types (Table 1) were evaluated. Normal skin samples from five healthy horses were also examined. Sections taken from paraffin blocks were stained by haematoxylin and eosin and re-evaluated to confirm the diagnosis. Histologically, the samples were characterised by epidermal hyperplasia with rete peg invading the dermal tissue beneath. A diffuse proliferation of dermal fibroblasts arranged in whorls and/or bundles was seen. All the tumour samples were diagnosed as equine sarcoids. All tumours were known to harbour BPV DNA [5]. Six out of 15 sarcoids and one normal skin sample were immediately frozen at −80°C until biochemical analysis.

**Table 1:** Presence of BPV-1 and immunohistochemical expression of BAG3 in equine sarcoids

Samples*	**Clinical type**	**BPV-1 DNA**	BAG3 immunohistochemical staining**
**T1**	Nodular	**+**	**+++**
**T2**	Mixed	**+**	**+/−**
**T3**	Verrucous	**+**	**++**
**T4**	Fibroblastic	**+**	**++**
**T5**	Flat	**+**	**+++**
**T6**	Verrucous	**+**	**++**
**T7**	Mixed	**+**	**++**
**T8**	Flat	**+**	**+/−**
**T9**	Fibroblastic	**+**	n.a.
**T10**	Verrucous	**+**	**+++**
**T11**	Verrucous	**+**	**+++**
**T12**	Nodular	**+**	**++**
**T13**	Verrucous	**+**	n.a.
**T14**	Nodular	**+**	**+**
**T15**	Mixed	**+**	**+**
**N1**	Normal skin	**+**	**+**
**N2**	Normal skin	n.a.	**+/−**
**N3**	Normal skin	n.a.	**+/−**
**N4**	Normal skin	n.a.	**+/−**
**N5**	Normal skin	n.a.	**+/−**

*T: tumour sample; N: normal skin sample.

**Immunoreactivity scoring was determined by two observers. The intensity of immune labelling for each specimen was scored on a four-tiered scale: +/−, very weak signal; +, weak signal; ++, moderate signal; +++, strong signal; n.a., not assessable.

### Immunohistochemistry

Fifteen sarcoid samples (T1-T15) and 5 normal skin samples (N1-N5) were stained. Briefly, paraffin sections were deparaffinised and blocked for endogenous peroxidase in 0.3% H_2_O_2_ in methanol for 20 min. Antigen enhancement was performed by pretreating with microwave heating (twice for 5 min each at 525 W). The anti-BAG3 antibody (AC-1 clone) was applied at 1:600 dilutions in phosphate-buffered saline (PBS) overnight at room temperature in a humidified chamber. The slides were washed three times with PBS and then incubated for 30 min with the appropriate biotinylated secondary antibody (labelled streptavidin–biotin (LSAB) Kit; DakoCytomation, Denmark) as previously reported [16]. Sections were washed three times with PBS and then incubated with streptavidin-conjugated to horseradish peroxidase (LSAB Kit; DakoCytomation, Denmark). Colour development was obtained by treatment with diaminobenzidine (DakoCytomation, Denmark) for 5 min. Sections were counterstained with Mayer’s haematoxylin. In the corresponding negative control section, the primary antibody was either omitted or replaced with appropriate normal serum. Due to technical issues associated with tissue loss during antigen retrieval, it was not possible to perform staining for BAG3 in 2 tumour samples (T9 and T13). The scoring of the immunoreactivity was determined in a “blind” study by two observers (GB and AC). The intensity of labelling in each specimen was scored from absent to very strong immunosignal.

### Cells and transfection

#### Cell lines

EqS04b cell line is a fully transformed sarcoid fibroblast line harbouring episomal BPV-1 genome. This line was a kind gift of Prof. L. Nasir (University of Glasgow, Scotland), who previously characterised and described it [17]. E. Derm fibroblast cell line, derived from horse dermis, and HeLa cells, human epithelioid cervix carcinoma cells expressing HPV-18 genome, were obtained from American Type Culture Collection (ATCC) (Manassas, VA, USA). All cells were maintained in DMEM (Biowhitaker, Lonza, NJ, USA) medium, supplemented with 10% (v/v) FBS, 2 mM L-glutamine and antibiotics, at 37°C in humidified 5% CO_2_ atmosphere. To ensure logarithmic growth, the cells were sub-cultured every three days.

#### Transfection

A specific small interfering RNA (siRNA) (5’-AAGGUUCAGACCAUCUUGGAA-3’) targeting *bag3* mRNA and a control, non-targeted (scr) RNA (5’-CAGUCGCGUUUGCGACUGG-3’) were obtained from Dharmacon (Thermo Fisher Scientific, Lafayette, CO, USA). EqS04b and E. Derm cells, at a density of 1 × 10^5^/mL, were transfected with siRNA and scrRNA at final concentration of 500 nM using lipofectamine™ RNAiMAX reagent (Invitrogen, Life Technologies, Grand Island, NY, USA). The cells were harvested at the time points indicated and BAG3 silencing was monitored in all the experiments by Western blotting. Human and equine siRNA targeting regions were compared (see Additional file 1) using Vista Browser 2.0 [18].

### Protein extraction and SDS PAGE/Western blotting

Six sarcoids (T1; T2; T3; T4; T5; T6) and one sample of normal skin (N1) were available for molecular analysis. These were snap frozen in liquid nitrogen and homogenised in ice-cold lysis buffer (50 mM Tris pH 7.5; 150 mM NaCl; 1 mM EDTA; 0.25% deoxycholic acid, 1% Triton X-100) added with 20 mM sodium pyrophosphate, 0.1 mg/mL aprotinin, 2 mM phenylmethylsulfonyl fluoride (PMSF), 10 mM sodium orthovanadate (Na_2_VO_3_), and 50 mM NaF. EqS04b, E. Derm and HeLa whole lysates for immunoblot analysis were prepared according to the standard protocol.

Tissue homogenates and cell lysates were clarified by centrifugation and protein concentration was determined by DC Protein Assay (Bio-Rad, Berkeley, CA, USA), using bovine serum albumin (BSA) as a standard. Proteins were fractionated on SDS-PAGE, transferred into nitrocellulose membranes, and immunoblotted with appropriate primary antibodies. Signals were visualised with appropriate horseradish peroxidase-conjugated secondary antibodies and enhanced chemiluminescence (Amersham Biosciences-GE Healthcare, NY, USA). Densitometry of bands was performed with ImageJ software (NHI, USA). The area under the curves, each relative to a band, was determined and the background was subtracted from the calculated values.

### Cell viability and drug treatment

Control and transfected cells, at a density of 1.2 × 10^5^/mL, were plated in 96 well-plates one day before the beginning of treatment with chemotherapeutics to allow cells to firmly adhere. Increasing doses of etoposide, tumour necrosis factor (TNF)-related apoptosis-inducing ligand (TRAIL), and phenethylisothiocyanate (PEITC) were added and plates were incubated for a further 24 h. At the end of incubation time, the number of viable cells was quantified by MTT ([3-(4,5-dimethylthiazol-2-yl)-2,5-diphenyl tetrazolium bromide]) assay. Absorption at 550 nm was assessed using a microplate reader (LabSystems, Vienna, VA, USA). MTT assay was also adopted to evaluate the effect of scrRNA and siRNA transfection on cell viability. In some experiments cell viability was also measured by Trypan Blue exclusion assay using a Bürker counting chamber.

### Cell cycle distribution and cell death analysis by flow cytometry

Cellular DNA content was evaluated by propidium iodide (PI) staining of permeabilised cells according to the protocol available [19]. Data from 5000 events per sample were collected. The percentages of the elements in the hypodiploid region and in G_0_/G_1_, S, G_2_/M phases of the cell cycle were calculated using the CellQuest or MODFIT software, respectively (Becton Dickinson, San Jose, CA, USA).

Apoptosis was determined by Human Annexin V/FITC kit (Bender MedSystem, Vienna, Austria) according to the manufacturer’s instructions. Green (Annexin V-FITC) and red (PI) fluorescence of individual cells were measured by flow cytometry. Electronic compensation was required to exclude overlapping of the two emission spectra.

### Analysis of PEITC-induced detachment

Culture media of scrRNA- and BAG3siRNA-transfected cells were changed 42 h following transfection to remove detached or died cells. PEITC or vehicle only were then added and cells were incubated for the times indicated. Non-transfected cells were included as an internal control. Phase contrast microscopy images were acquired after 6 h incubation. A parallel set of plates was incubated for a further two hours and used, after carefully removing media, to measure the number of still attached cells by MTT assay.

### Microscopy analysis

Phase contrast microscopy analysis was performed using a Leica DM IL LED microscope (Leica Microsystems, Wetzlar, Germany) equipped with a 40 × objective and images were acquired from randomly selected fields.

### Statistical analysis

Unless otherwise specified, data reported in each figure are the mean value ± S.E.M. of at least three experiments performed in duplicate. Differences between treatment groups were analysed by the student *t* test. Differences were considered significant when *p* < 0.05.

## Results

### BAG3 expression in equine sarcoids

**Figure 1:**
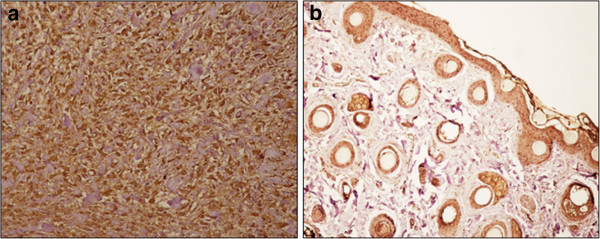
**BAG3 expression in equine sarcoid and normal fibroblasts. (a)** Neoplastic fibroblasts from equine sarcoid show strong immunoreactivity for BAG3. Streptavidin-biotin peroxidase method. Mayer’s haematoxylin nuclear counterstain ×240. **(b)** Dermal fibroblasts from normal equine skin are weakly immunostained for BAG3. Streptavidin-biotin peroxidase method. Mayer’s haematoxylin nuclear counterstain ×120.

We analysed immunohistochemically 15 tumour samples and 5 normal skin samples for BAG3 expression (Table 1). Thirteen out of 15 tumour samples stained positive for BAG3 (85%). Four out of 13 samples (30%) showed strong immunoreactivity; 5 out of 13 (40%) displayed a moderate immunostaining signal; the remaining sarcoid samples, showed a weak (15%) and a very weak (15%) immunostaining signal for BAG3 throughout the lesions. In the positive samples the immunoreactivity was observed in almost all neoplastic fibroblasts (Figure 1a). Normal dermal fibroblasts derived from healthy horses displayed very weak staining patterns for BAG3 expression (Figure 1b). Samples (T1-T6 and N1) were analysed biochemically and the anti-BAG3 antibody recognised a band of the expected molecular weight in all samples examined (data not shown).

### BAG3 down-modulation in equine cell lines

**Figure 2:**
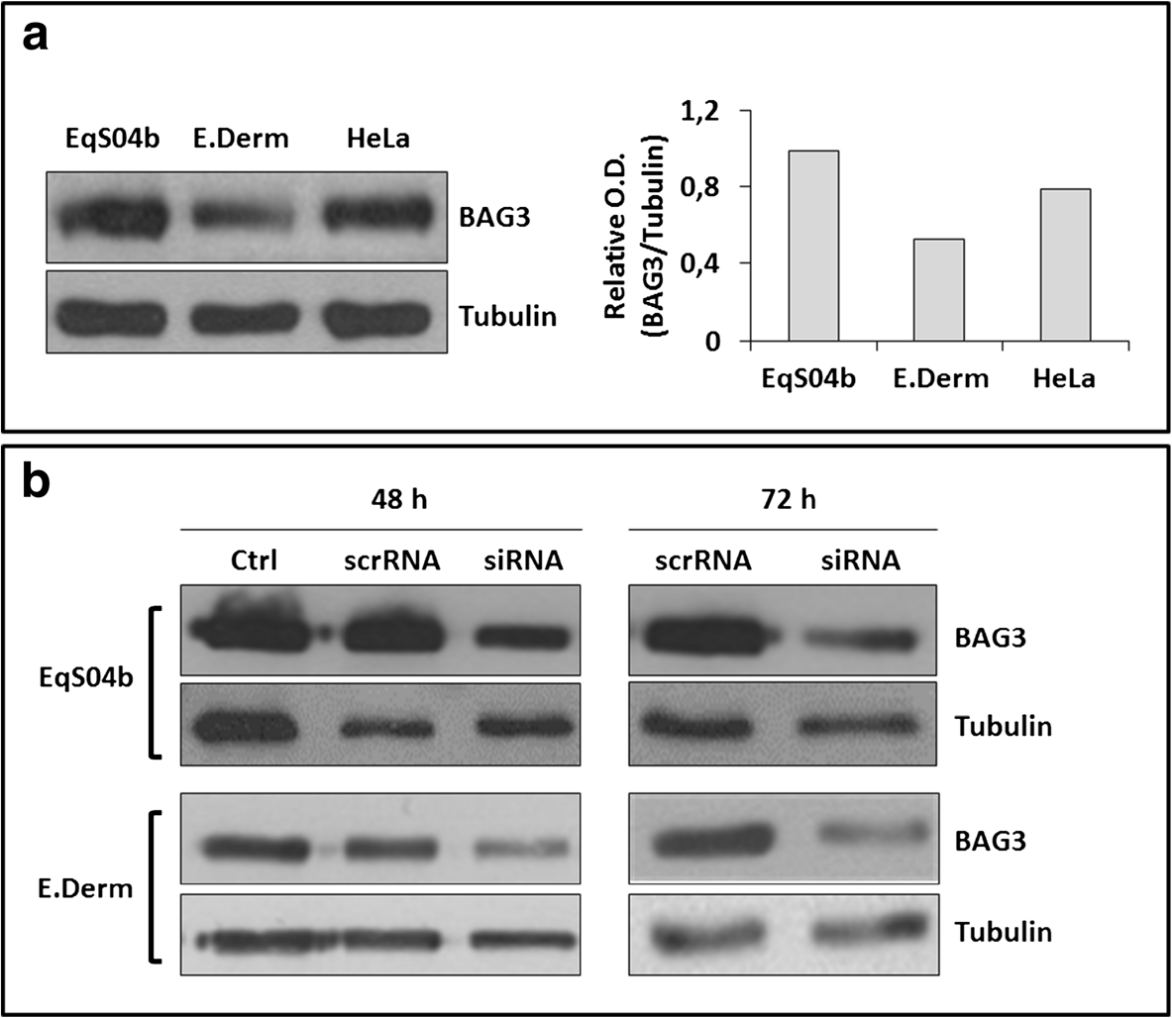
**BAG3 expression and down-modulation in equine and human cell lines. (a)** BAG3 expression in equine cell lines. EqS04b, E. Derm and human HeLa cell total extracts were analysed by Western blotting for BAG3 levels. The blots were also probed for tubulin as the loading control. Blot is from one experiment representative of at least two with similar results. On the right: densitometry analysis of bands. **(b)** BAG3 down-modulation in EqS04b and E. Derm cells. EqS04b and E. Derm cells were transfected with BAG3siRNA (siRNA) or a non-targeting siRNA (scrRNA). Non-transfected cells were included as controls (Ctrl). At the times indicated, total cell extracts were prepared and analysed by Western blotting for BAG3 levels. The blots were also probed for tubulin as the loading control. Blots are from one experiment representative of at least two with similar results.

BAG3 expression was also monitored in whole lysates of EqS04b and E. Derm cells by Western blotting. Human HeLa cells were included as a control [20]. As shown in Figure 2a, BAG3 protein signal was successfully detected in both equine cell lines. Notably, the levels of BAG3 were higher in a sarcoid-derived EqS04b cell line than in E. Derm cells, taken as the normal counterpart.

Since BAG3 anti-apoptotic effect in human tumour cell lines has been well established [8,9], we aimed at evaluating whether BAG3 might have a pro-survival role also in EqS04b and E. Derm cells. To this end, BAG3 protein expression was down-modulated by using a BAG3-specific siRNA (BAG3siRNA) and cell viability was monitored. Cells transfected with scrambled RNA (scrRNA) and non-transfected cells were included as controls. Figure 2b shows that reduction of BAG3 levels in siRNA-transfected cells was already detectable at 48 h, becoming more evident at 72 h. However, complete BAG3 knock-down failed, even using a BAG3siRNA concentration two fold higher than that used for BAG3 full silencing in human cells [9].

**Figure 3:**
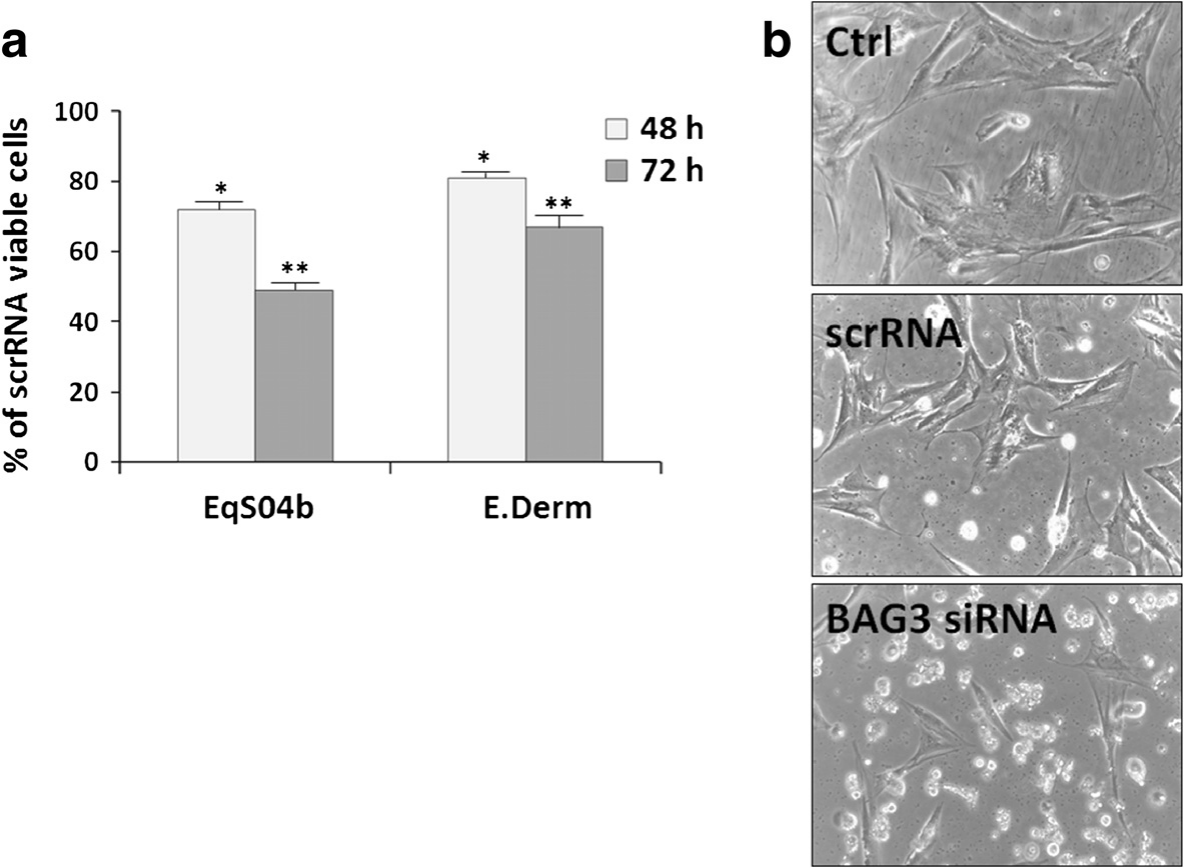
**Effect of BAG3 down-regulation on cell viability and morphology. (a)** Number of viable cells in BAG3-silenced EqS04b and E. Derm cells at 48 h and 72 h following transfection. Data, expressed as a percentage of the number of scrRNA-transfected viable cells measured at the same times, are the mean values ± S.E.M. from at least three experiments (*p < 0.05, **p < 0.001 versus scrRNA-treated cells); **(b)** Phase contrast microscopy images of non-transfected (Ctrl), scrRNA- and BAG3siRNA (siRNA)-transfected EqS04b samples at 56 h after transfection. Images are from one analysis representative of at least three experiments with similar results.

Then, we evaluated the effect of BAG3 silencing on cell number (Figure 3a). The number of EqS04b viable cells, at 48 h and 72 h following BAG3siRNA transfection, was about 72% and 51%, respectively, of that in scrRNA-transfected cells. A time-dependent decrease of cell number, even though less marked, was also observed in the E. Derm cell line. It should be underlined that transfection procedure caused *per se* a slight reduction of cell number in both cell lines probably due to a loss of attachment efficiency. In particular, the number of scrRNA-treated EqSO4b and E. Derm cells was, irrespective of the times following transfection, about 11% ± 1.3 and 13% ± 1.6, respectively, lower than in their relative non-transfected controls.

Light microscopy images in Figure 3b revealed that BAG3siRNA transfection caused most of the EqS04b cells to acquire a round shaped morphology, ultimately resulting in cell detachment and death. Similar results were also obtained, even though less marked, in BAG3siRNA-transfected E. Derm cells (data not shown).

### BAG3 silencing induces apoptosis and cell cycle arrest

Since in preliminary experiments we found that BAG3 silencing elicited qualitatively, but not quantitatively, similar effects in both cell lines, we detail below only the results obtained with EqS04b cells.

**Figure 4:**
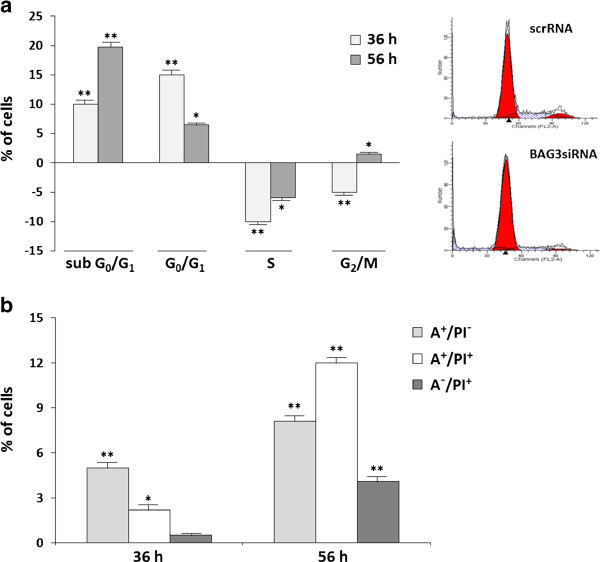
**BAG3 down-regulation induces apoptosis and affects cell cycle progression in EqS04b cells. (a)** On the Y axis, the percentages of hypodiploid cells (subG_0_/G_1_) and of cells in each cell cycle phase of BAG3siRNA-transfected EqS04b cells subtracted for the corresponding percentages in scrRNA-transfected cells. Data presented are the mean values ± S.E.M. from at least three experiments (*p < 0.05, **p < 0.001 calculated on raw data of BAG3siRNA-silenced versus scrRNA-treated cells). Mean values (%) ± S.E.M. in scrRNA-transfected cells: i) at 36 h, subG_0_/G_1_ 4.9 ± 1.2; G_0_/G_1_ 68.59 ± 3.7; S 21.08 ± 1.8; G_2_/M 9.03 ± 1.0; ii) at 56 h, subG_0_/G_1_ 3.5 ± 1; G_0_/G_1_ 66.8 ± 2.3; S 22.4 ± 2.1; G_2_/M 10.6 ± 1.1. Representative histograms of cell cycle profiles of scrRNA- and BAG3siRNA-transfected cells at 36 h are on the left. **(b)** On the Y axis, the percentages of Annexin V positive (A^+^/PI^-^, early apoptotic cells; A^+^/PI^+^, late apoptotic cells) and necrotic cells (A^-^/PI^+^) in BAG3siRNA-silenced EqS04b cells subtracted for the corresponding percentages in scrRNA-transfected cells. Data presented are the mean values ± S.E.M. from at least three experiments (*p < 0.05, **p < 0.001 calculated on raw data of BAG3siRNA-silenced versus scrRNA-treated cells). Mean values (%) ± S.E.M. in scrRNA-transfected cells: i) at 36 h, A^+^/PI^-^, 4.2 ± 1.1; A^+^/PI^+^, 2.3 ± 0.96; A^-^/PI^+^, 3.1 ± 1; ii) at 56 h the values became comparable to those of non-transfected control cells (≤ 2% in A^+^/PI^-^, A^+^/PI^+^, A^-^/PI^+^ gated quadrants).

EqS04b transfected with BAG3siRNA were collected at 36 h and 56 h following transfection and were stained under permeabilising conditions with propidium iodide (PI), a DNA intercalating agent. They were then analyzed by flow cytometry to discriminate cell populations on the basis of their DNA content. ScrRNA-transfected cells were included as controls. Data summarised in Figure 4a show that BAG3 down-modulation caused a time-dependent increase of cells with a subG_0_/G_1_ DNA content (< n, hypodiploid cells), indicative of the apoptotic mode of cell death. In addition, the percentages of BAG3-silenced cells in G_0_/G_1_ (DNA content = n) were significantly higher, especially at 36 h, than in scrRNA-transfected cells. Because of the high percentage of subG_0_/G_1_ cells at 56 h, we could argue that the less marked cell cycle impairment observed at that time could be ascribed to apoptotic events occurring mainly in cells previously accumulated in G_0_/G_1_, rather than to a recovery of normal cell cycle progression.

Next, because cells integrated as subG_0_/G_1_ may include not only frankly apoptotic cells, but also necrotic fragments, we took advantage of Annexin V/PI assay to discriminate between the two different modes of cell death. BAG3siRNA- and scrRNA-treated EqS04b cells were double stained, under non-permealizing conditions, with Annexin V, which binds phosphatidyl serine (PS) residues exposed on membranes of apoptotic cells, and PI, which can penetrate only in cells with compromised plasma membrane, such as late apoptotic and necrotic cells. Data summarised in Figure 4b show a time-dependent increase of PS positive cells (Annexin V^+^/PI^-^, early apoptosis and Annexin V^+^/PI^+^, late apoptosis) in BAG3siRNA- respect to scrRNA-transfected EqS04b cells. A switch from an apoptotic to a necrotic mode of cell death was observed only at 56 h following BAG3siRNA transfection. These findings indicate that BAG3 down-regulation promoted equine cell death primarily by apoptosis.

### BAG3 down-modulation sensitizes equine cell lines to chemical-induced toxicity

Next we aimed at verifying whether BAG3 down-regulation, in addition to sensitising cells to basal apoptotic signals, might also increase EqS04b and E. Derm response to drugs, known to promote apoptosis in human tumour-derived cell lines. First of all, we characterised EqS04b and E. Derm cells for their response to three pro-apoptotic chemicals, each acting by a different main mechanism. In particular, we tested etoposide, an inhibitor of topoisomerase II [21], TRAIL, which activates extrinsic pathway of apoptosis [22], and PEITC, which promotes a mitochondrial-mediated intrinsic pathway of apoptosis [23]. Human HeLa cells were included in the experiments. Cells were exposed to increasing doses of each chemical and, after 24 h incubation, the number of cells was measured by MTT assay. The Ic50 values (the amount of drug required to inhibit cell growth by 50%) in the three cell lines were calculated from the respective dose–response curves (Table 2). EqS04b and, to a lower extent, E. Derm cells were markedly more resistant to etoposide and TRAIL than human HeLa cells. Conversely, both equine cell lines were highly susceptible, with E. Derm displaying the highest susceptibility, to PEITC.

**Table 2:** **Cell growth inhibition potency (Ic50**_**24 h**_**) of chemotherapeutics in equine and human cells**

**Cell line**	**Ic50**_**24 h **_**(μM)**		
	**Etoposide**	**TRAIL**	**PEITC**
**EqS04b**	150 ±12*	>1500 ng/mL	25 ± 1.9
**E. Derm**	80 ± 10	>1000 ng/mL	19 ± 1.5
**HeLa**	30 ± 2.2	30 ng/mL	12.5 ± 0.9

* Mean values ± S.E.M.

**Figure 5:**
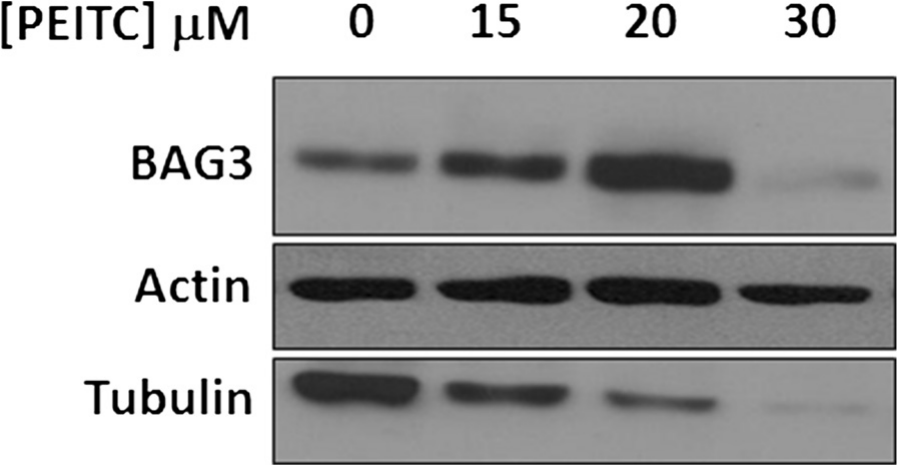
**PEITC modulates BAG3 protein levels*.*** Cell extracts from EqS04b cells exposed to the indicated doses of PEITC for 12 h were monitored for BAG3 levels. Actin levels were checked as the loading control. Filters were also probed for PEITC-induced tubulin degradation. Blot is from one experiment representative of at least two with similar results.

To assess whether BAG3 might play some roles in equine cell response to PEITC, we monitored the effect of increasing PEITC doses on BAG3 protein levels. Blots reported in Figure 5 show that EqS04b exposure for 12 h to PEITC doses lower than the Ic50 value led to increased BAG3 protein expression. Conversely, BAG3 levels were dramatically reduced at 30 μM PEITC, a dose at which cells were already committed towards an irreversible death fate. Modulation of BAG3 expression by PEITC was also observed in the E. Derm cell line (data not shown). The blot in Figure 5 was re-probed for tubulin, taken as a marker of PEITC effectiveness. In preliminary experiments, we, indeed, confirmed that PEITC promotes tubulin degradation in equine cell lines as previously demonstrated in human cell lines [24,25].

**Figure 6:**
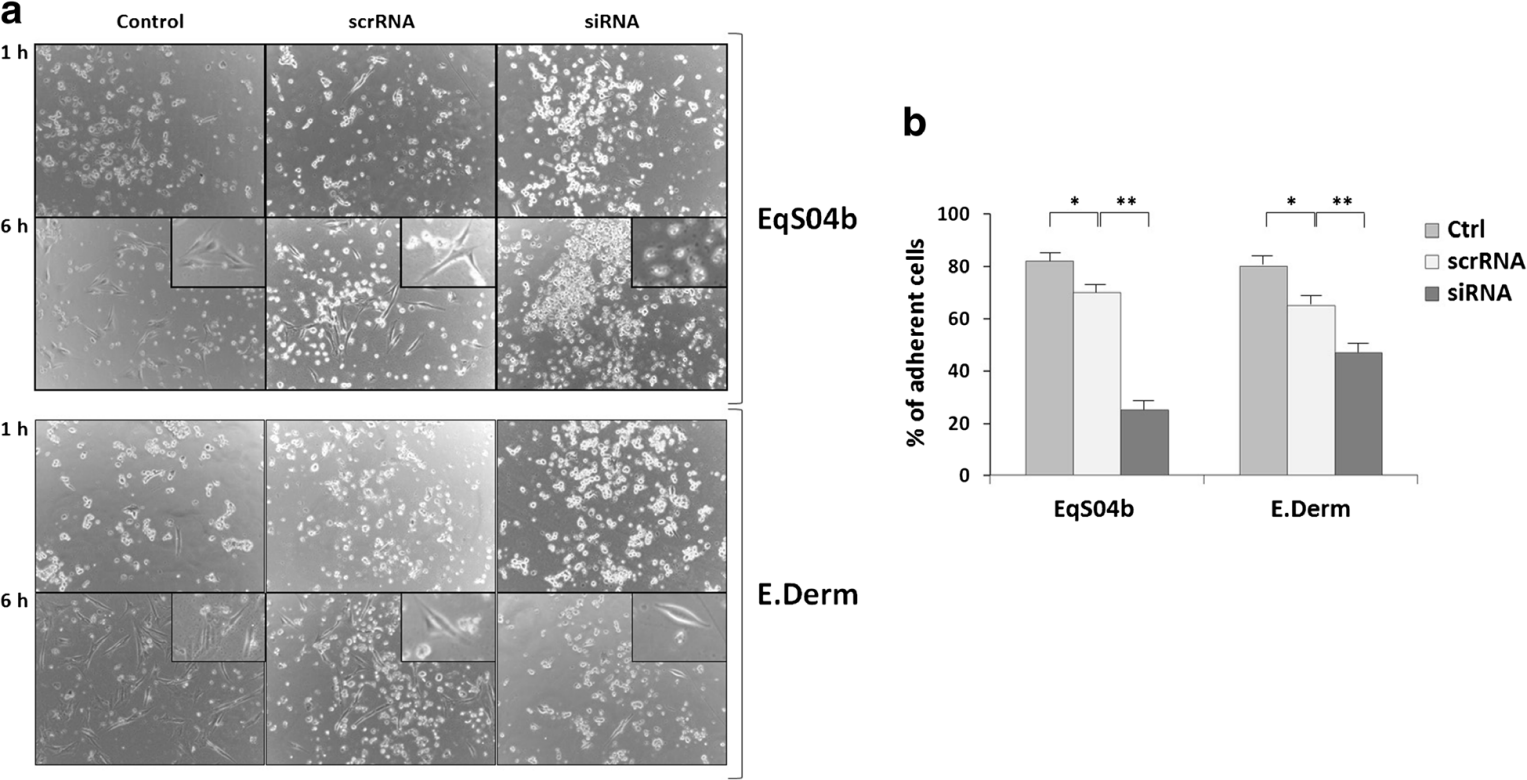
**BAG3 down-modulation sensitizes equine cell lines to PEITC-induced detachment.** Control, scrRNA- and BAG3siRNA-treated cells were exposed to PEITC 42 h after transfection. Control and scrRNA-treated (scrRNA) EqS04b and E. Derm cells were exposed to 20 μM and 15 μM PEITC, respectively, while EqSO4b and E. Derm BAG3 silenced cells (siRNA) were exposed to 15 μM and 12 μM PEITC, respectively, in view of the lower number of cells (about 70-80% of control and scrRNA cells). **(a)** Phase contrast images of control (left panels), scrRNA-transfected (middle panels), and BAG3siRNA (siRNA)-transfected (right panels) cells were acquired at the times indicated (images from one experiment representative of three with similar results). **(b)** Percentages of adherent cells at 8 h following PEITC treatment in non-transfected (ctrl), scrRNA-transfected, and BAG3siRNA (siRNA)-transfected cells respect to their corresponding controls exposed to vehicle only. Data presented are the mean values ± S.E.M. from at least three experiments (*p < 0.05, **p < 0.001; scrRNA-transfected versus non-transfected or siRNA versus scrRNA).

The observed BAG3 modulation by PEITC prompted us to investigate whether BAG3 down-regulation could actually lead to an increase of EqS04b and E. Derm susceptibility to the chemical. We carefully designed an experimental plan to minimise the risk of false positive results. In particular, we adopted the following experimental conditions: i) PEITC was added at 42 h following transfection, a time at which the number of BAG3-silenced viable EqS04b and E. Derm cells was reduced by only 25% and 20%, respectively, compared to the corresponding scrRNA-transfected cells; ii) to take into account such a reduction, the doses of PEITC added to BAG3-silenced EqS04b and E. Derm cells were 25% and 20%, respectively lower than those used for the corresponding non-transfected and scrRNA-transfected controls. Moreover, to completely avoid overlap between PEITC-induced toxicity and BAG3 silencing–induced cell death, we took advantage of PEITC short-term treatment-induced cellular morphological changes. In particular, treatments of non-transfected EqS04b and E. Derm cells with PEITC doses lower than the respective Ic50 values for 1 h caused cells to acquire a round shaped morphology, but already after 6 h of incubation, the cells were able to restore multiple contacts with the extracellular matrix and spread (Figure 6a, left panels). Quite similar results were obtained when we monitored the effect of PEITC short-term treatment on scrRNA-transfected cells. Cells were still able to recover an elongated fibroblastoid shape, but less efficiently than control cells, possibly because the transfection procedure may *per se* affect membrane structure/permeability to PEITC (Figure 6a, middle panels). Conversely, microscopy analysis of BAG3-silenced cells clearly showed that most of the PEITC-treated cells remained round shaped, aggregated and detached (Figure 6a, right panels). Remarkably, PEITC-induced detachment seemed less marked in BAG3-silenced E. Derm cells than in EqS04b. These results were taken as evidence, even if only qualitative, of an increased susceptibility of EqS04b and, less markedly, of E. Derm cells to PEITC upon BAG3 down-modulation.

To confirm and quantify the results obtained by phase microscopy analysis, we performed an *ad hoc* experiment. At 42 h following transfection, scrRNA- and BAG3siRNA-transfected cells were treated according to the above procedure with PEITC or vehicle only. After 8 h incubation, media, containing floating cells, were carefully removed to measure still adherent cells by MTT assay. The number of cells in PEITC-treated samples is expressed as the percentage of that in vehicle–treated respective controls. Data summarized in Figure 6b confirmed microscopy results. In fact, scrRNA-transfected EqS04b and E. Derm resulted more susceptible, even slightly, to PEITC than non-transfected cells. A largely more marked decrease of cell number occurred in BAG3-silenced cells exposed to PEITC. Notably, while in E. Derm the percentage of attached cells was about 50% of the corresponding vehicle-treated control, in EqS04b the value dropped to about 25%, thus unequivocally demonstrating that BAG3 efficiently counteracts, especially in BPV-1 positive cell death signals triggered by PEITC. These results were qualitatively confirmed by microscopic counting, upon concentration by centrifugation, of floating cells in collected media (data not shown).

## Discussion

In human cancer cells, BAG3 protein is known to sustain cell survival [8,26]. Overexpression of *bag3* promotes, indeed, survival whereas its down-regulation sensitizes cells to apoptosis, both in vitro and in vivo [8,9,26]. In the present study, for the first time, we demonstrate that BAG3 protein is expressed in a subset of naturally occurring equine sarcoids, expressing BPV-1 genome, and that it sensitizes equine sarcoid-derived cells to PEITC, a promising cancer chemopreventive/chemotherapeutic agent present in edible cruciferous vegetables [27].

The first evidence of a possible involvement of BAG3 in equine sarcoid carcinogenesis was obtained by immunohistochemistry analysis of tumour samples. We found that 13 out of 15 tumour samples stained, even though to a different grade, positive for BAG3. Notably, normal dermal fibroblasts derived from healthy horses displayed a very weak staining pattern for BAG3 expression. Sarcoids may exist as six different clinical types. We did not find any correlation between overexpression of BAG3 and clinical appearance, suggesting the existence of a common mechanism underlying the up-regulation of protein expression, that acts early during the development of equine sarcoids.

To get more insight into a possible pro-survival role of BAG3 in sarcoids, biochemical studies were conducted on EqS04b cells, an equine sarcoid-derived BPV-1 positive cell line [17], as an in vitro model. We found that EqS04b cells express higher levels of BAG3 than E. Derm cells, taken as the normal counterpart. Moreover, by means of siRNA technology, we demonstrated that BAG3 down-regulation induced, more markedly in EqS04b than in E. Derm cells, apoptotic death and cell cycle arrest in G_0_/G_1_. These findings support the idea of some cooperation between BAG3 and viral oncoproteins in sustaining cell survival and proliferation.

Because BAG3 has been shown to underlie resistance to chemotherapy [28], and considering that, currently, there is no 100% effective therapy for the treatment of equine sarcoids [3], we would investigate whether the lack of BAG3 function might specifically sensitize BPV-1 positive EqS04b cells to potentially chemotherapeutic agents.

The analysis of the basal response to etoposide and TRAIL, two well known anti-tumour chemicals in humans [29], showed that both equine cell lines were very low, if not, susceptible to these cell-killing agents. In particular the lack of any significant response to TRAIL could be explained on the basis of dissimilarity between equine and human death receptors, DR4 (TRAIL-RI) and DR5 (TRAIL-RII). In fact, comparison between human and equine receptor sequence by compositional matrix adjust method showed identities = 43%, positives = 54%, gaps = 17% [18]. Conversely, we found that both equine cell lines were highly susceptible to PEITC. Interestingly, EqS04b displayed lower susceptibility to PEITC, as well as to etoposide, than E. Derm cells thus supporting the hypothesis that the BPV genome present in EqS04b provides some protection against exogenous death stimuli. This hypothesis was also supported by other studies on BPV-induced bovine urinary bladder tumours in which high levels of E5 were found to correlate with BAG3 overexpression (F. Roperto, personal communication). However, our data seem to be in contrast with those reported in the study of Finlay et al. [30], who provided evidence that the presence of the BPV-1 genome is, rather, associated to increased sensitivity of equine sarcoid-derived cells to UVB- and cisplatin-induced apoptosis. Such a discrepancy between their and our findings might possibly be due to the use of different cell-killing agents, which, acting by different mechanisms, may interfere with specific cell death/survival signalling pathways.

Subsequent experiments demonstrated that BAG3 is a key component of the multifaceted pro-survival machinery activated by equine cells as a primary response to PEITC pro-death signals. In fact, exposure of cells to low PEITC doses (lower than Ic50) caused an increase of BAG3 levels. On the contrary, at highly cytotoxic PEITC doses (higher than the Ic50 value) BAG3 levels were reduced thus suggesting that down-regulation of BAG3 was a prerequisite for commitment of cells towards an irreversible death fate. Targeting BAG3, which is known to prevent mitochondrial cytochrome *c* release [31] and apoptosome assembly [8], allowed PEITC to activate mitochondrial-mediated apoptosis.

At least, by siRNA technology we unequivocally demonstrated the role of BAG3 in counteracting PEITC-triggered pro-apoptotic signals. In fact, BAG3 down-modulation sensitised equine cells to PEITC-induced cell detachment and subsequent cell death. The central role of BAG3 in cell adhesion pathways and, consequently, in tumour invasion and metastasis, has been previously demonstrated in human epithelial cancer cells [32,33]. Remarkably, the observed protective role of BAG3 against PEITC-promoted cell detachment and death resulted largely more relevant in BPV-1 infected cells. In fact, the increased susceptibility to PEITC upon BAG3 silencing was in EqS04b cells about two fold higher than in E. Derm cells. This result could be taken as a further support of the presence of some positive cooperation between BAG3 and BPV-1 oncoproteins.

It could be hypothesised that BAG3 is effective in preventing HSP70-mediated delivery to proteasome [34] of BPV-1 oncoproteins. Indeed, BAG3-HSP70 interaction in virus-infected human cells has been documented [14,15]. It has been shown that BAG3 co-localizes in the nucleus of melanoma cells with HSC70/HSP70, HSP90 and ORF29p, a LAP (latency associated protein) produced by Varicella Zoster Virus [14]. Moreover, it is worthwhile noting that HSP70 was found as a molecular partner of BPV E7 oncoprotein in sarcoid biopsies (manuscript in preparation). These examples of interaction with viral proteins and/or involvement of BAG3 in viral replication pathways suggest that also in our system BAG3 could someway interact with BPV-1 oncoproteins, thus sustaining EqSO4b BPV-1 positive cell survival.

We cannot exclude, however, that BPV-1 oncoproteins are, rather, responsible for overexpression of anti-apoptotic proteins, such as BAG3, in sarcoids. It is anecdotically reported that sarcoids arise in body areas where irritation/inflammation occurs. Since BAG3 gene expression is induced by stressful agents, through the activation of heat shock factor (HSF-1) [9], we could also speculate that a BAG3 increase is likely part of cell response to injury, which, in turn, triggers/sustains BPV viral oncoprotein expression.

In conclusion, this study reveals, for the first time, the high susceptibility of sarcoid-derived BPV-1 positive EqS04b cell line to the natural compound PEITC, and highlights the role of BAG3 protein to protect these cells against PEITC-triggered pro-death signals. Thus our findings, indicating BAG3 as a potential therapeutic target, may provide useful information to identify appropriate and efficient therapeutic strategy for bovine papillomavirus infections.

## Abbreviations

BPV: Bovine Papilloma Virus; HPV: Human Papilloma Virus; TRAIL: Tumour necrosis factor (TNF)-related apoptosis-inducing ligand; PEITC: phenethylisothiocyanate.

## Competing interests

The authors declare that they have no competing interests.

## Authors’ contributions

The work presented here was carried out in collaboration between all authors. MAB conceived the biochemical study design, coordinated the experiments*,* and drafted the manuscript. RC designed most of the biochemical in vitro methods and experiments, carried out the laboratory experiments, analysed the data, and interpreted the results*.* MD contributed to transfection experiments. MD and DG performed statistical analysis and participated in a literature search, and presentation of data. GA and AC performed immunohistochemical and biochemical studies on equine sarcoid samples. GB and FR conceived and coordinated the study on equine sarcoids, contributed to draft the manuscript and to critically revise it. All authors read and approved the final manuscript.

## Supplementary Material

Additional file 1 **Comparison of human and equine siRNA targeting regions.** Comparative sequence analysis of human and horse bag3 mRNA sequence (NM_004281.3 GI:62530382 and XM_001496279.3 GI:338716418) was performed by using VISTA Browser tool [18]. Homo sapiens sequence was selected as the reference so the level of conservation between this reference and horse (equus caballus) bag3 mRNA sequence was displayed. Conserved regions are highlighted under the curve, with different colors used for coding (purple) and noncoding sequences (grey), default values for conservation cutoff (X% over Y bp) were used. A zoom of the siRNA targeting region is displayed (in the red circle) and the arrow shows the one base mismatch between the human sequence (siRNA target sequence) and the horse sequence. Click here for file
