# Supplementary figures and images for: BAG3 protects Bovine Papillomavirus type 1-transformed equine fibroblasts against pro-death signals
Source: Vet Res. 2013 Jul 22;44(1):61. doi: 10.1186/1297-9716-44-61 (PMC3729419; doi:10.1186/1297-9716-44-61)

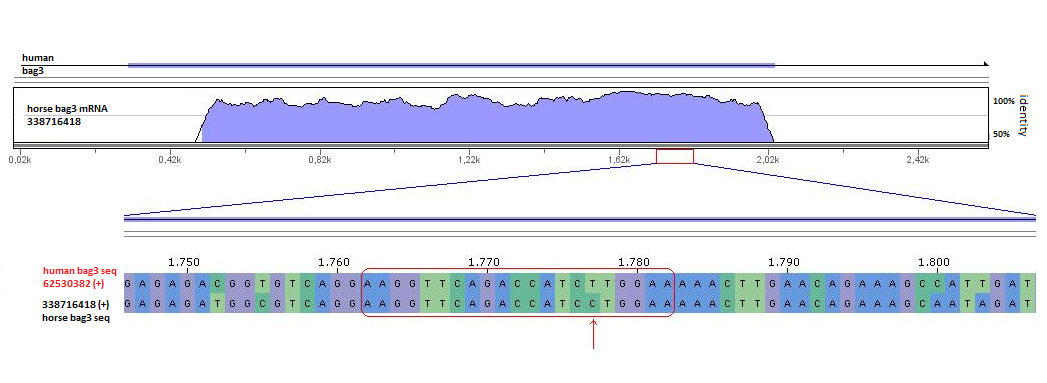

Supplement: Additional file 1 — Comparison of human and equine siRNA targeting regions. Comparative sequence analysis of human and horse bag3 mRNA sequence (NM_004281.3 GI:62530382 and XM_001496279.3 GI:338716418) was performed by using VISTA Browser tool [18]. Homo sapiens sequence was selected as the reference so the level of conservation between this reference and horse (equus caballus) bag3 mRNA sequence was displayed. Conserved regions are highlighted under the curve, with different colors used for coding (purple) and noncoding sequences (grey), default values for conservation cutoff (X% over Y bp) were used. A zoom of the siRNA targeting region is displayed (in the red circle) and the arrow shows the one base mismatch between the human sequence (siRNA target sequence) and the horse sequence. [file 1297-9716-44-61-S1.tiff]
